# Chemical, Physiological and Molecular Responses of Host Plants to Lepidopteran Egg-Laying

**DOI:** 10.3389/fpls.2019.01768

**Published:** 2020-01-30

**Authors:** Cinzia Margherita Bertea, Luca Pietro Casacci, Simona Bonelli, Arianna Zampollo, Francesca Barbero

**Affiliations:** ^1^Plant Physiology Unit, Department of Life Sciences and Systems Biology, Turin University, Turin, Italy; ^2^Zoolab, Department of Life Sciences and Systems Biology, Turin University, Turin, Italy; ^3^Museum and Institute of Zoology, Polish Academy of Sciences, Warsaw, Poland

**Keywords:** butterflies, moths, egg-associated microorganisms, interactions, elicitors

## Abstract

Plant-lepidopteran interactions involve complex processes encompassing molecules and regulators to counteract defense responses they develop against each other. Lepidoptera identify plants for oviposition and exploit them as larval food sources to complete their development. In turn, plants adopt different strategies to overcome and limit herbivorous damages. The insect egg deposition on leaves can already induce a number of defense responses in several plant species. This minireview deals with the main features involved in the interaction between plants and lepidopteran egg-laying, focusing on responses from both insect and plant side. We discuss different aspects of direct and indirect plant responses triggered by lepidopteran oviposition. In particular, we focus our attention on the mechanisms underlying egg-induced plant defenses that can i) directly damage the eggs such as localized hypersensitive response (HR)-like necrosis, neoplasm formation, production of ovicidal compounds and ii) indirect defenses, such as production of oviposition-induced plant volatiles (OIPVs) used to attract natural enemies (parasitoids) able to kill the eggs or hatching larvae. We provide an overview of chemical, physiological, and molecular egg-mediated plant responses induced by both specialist and generalist lepidopteran species, also dealing with effectors, elicitors, and chemical signals involved in the process. Egg-associated microorganisms are also discussed, although little is known about this third partner participating in plant-lepidopteran interactions.

## The Insect Side: How Lepidoptera Use Plant Signals to Select Oviposition Sites

Lepidoptera mainly depend on plants to complete their development. The choices of gravid females for a suitable oviposition site will severely affect their offspring performances, thus impacting the whole population's survival (García-Barros and Fartmann, 2009). The allocation of eggs on specific larval host plants (LHPs) could be determined by a dynamic hierarchy of biotic and abiotic factors (Carrasco et al., 2015). Not only the plant species and its quality, but also the microclimatic conditions in the surroundings, the intra- or interspecific brood competition, and the occurrence of symbionts or predators might regulate egg-laying behavior in Lepidoptera (Renwick and Chew, 1994; Ghidotti et al., 2018).

Females searching for an ideal LHP have to combine multifarious sensory information mainly made of chemical, visual, or tactile stimuli (Brévault and Quilici, 2010). Strategies and signals involved are extremely variable and can be summarized as follows: (i) blends of plant volatiles and (ii) visual cues enhance the flight towards the oviposition site and reveal where to land, (iii) substrate compounds are assessed using legs, ovipositor, or proboscis and function as proxies for quality and suitability of the plant site (Reisenman et al., 2010).

Although plants benefit from attracting pollinators, the majority of butterflies and moths should be considered foes as their larvae can be voracious herbivores. Thus, there is a trade-off between resources employed by plants to attract insects for their reproduction and those used to repel enemies. Wounds, bites, or the simple glueing of eggs are signs of current or future herbivore threat and can trigger striking chemical, physiological, and systemic reactions in plants (revised by Hilker and Fatouros, 2015; Schuman and Baldwin, 2016). If constitutive plant compounds usually act as attractants, blends of chemicals released as deterrents to eggs or herbivores may signal a resource already occupied. According to the lepidopteran species, the presence of conspecifics or heterospecifics could enhance (e.g., Anderson and Alborn, 1999) or deter (Sato et al., 1999; De Moraes et al., 2001) oviposition behavior.

Whatever the outcome (i.e., attraction or deterrence), the presence of prior egg deposition is detected by females not exclusively through sight or the perception of oviposition deterring pheromones, such as those released by *Pieris* spp. (Schoonhoven et al., 1990) or *Anthocharis cardamines* (Dempster, 1992), but also by discriminating oviposition-induced plant volatiles (OIPVs; see further section). For instance, by perceiving OIVPs released by *Brassica nigra*, *Pieris brassicae* selects egg-free plants as oviposition sites (Fatouros et al., 2012).

**Figure 1:**
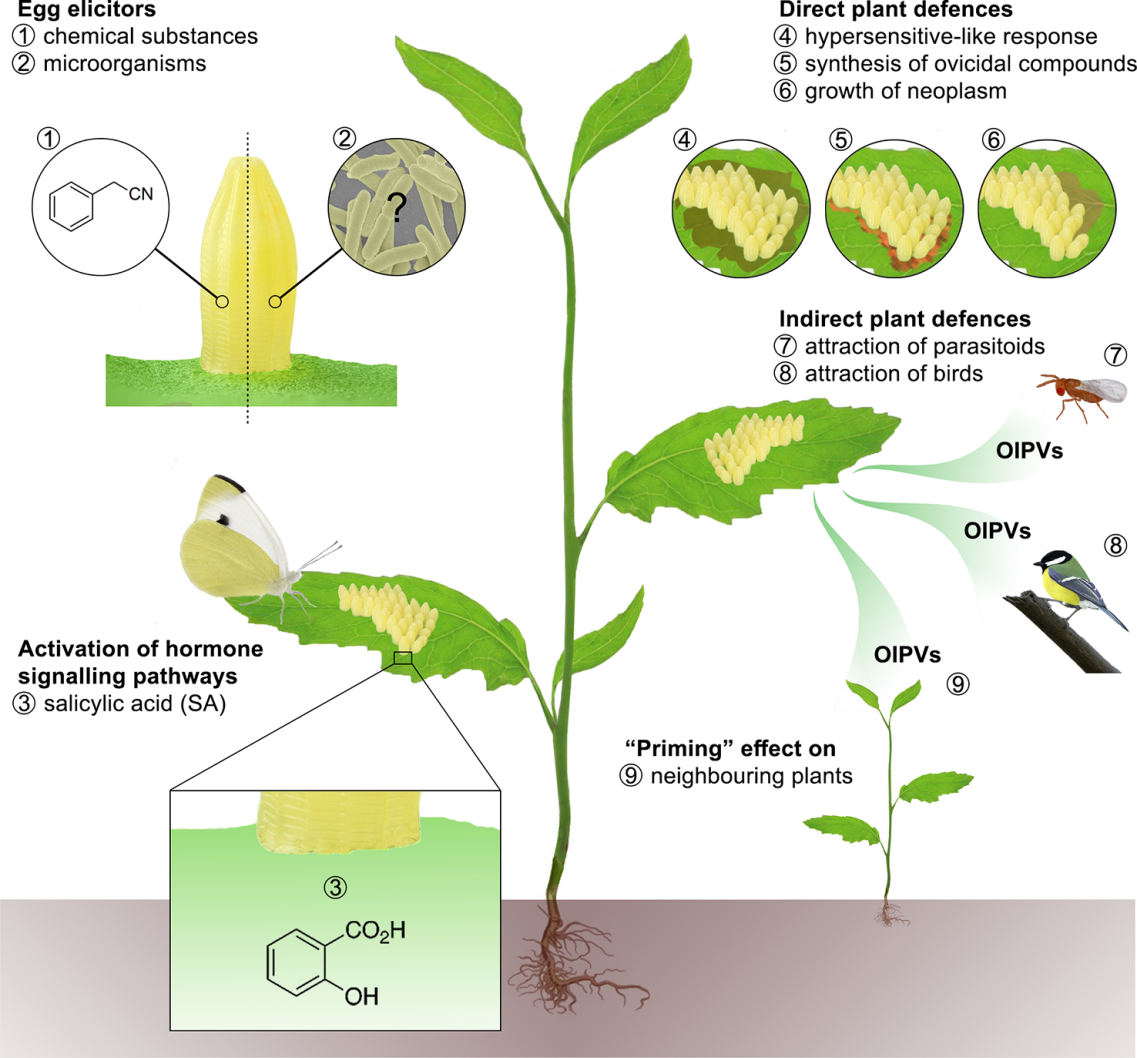
Lepidopteran oviposition could represent a potential risk for host plants (Hilker and Fatouros, 2015), which can activate a pre-empted defense strategy to prevent or limit significant injuries. Therefore, plants have developed the ability to use egg deposition as a warning cue to increase defenses against larvae after hatching (Beyaert et al., 2011) or even modify their own phenology to achieve an early flowering and reproduction (Lucas-Barbosa et al., 2013). Indeed, there is a bulk of evidence on the existence of specific plant responses that may endeavor to damage eggs directly or indirectly. Egg elicitors, i.e. 1) chemical substances present on the egg surface (e.g. benzyl cyanide), and possibly 2) egg-associated microorganisms trigger downstream defense responses regulated through hormone signaling pathways of which 3) salicylic acid (SA) plays a pivotal role (Hilfiker et al., 2014). Direct defense strategies include 4) necrotic tissue (HR-like necrosis), 5) ovicidal compounds (H_2_O_2_) (Geuss et al., 2017) or 6) callose formation. Lepidopteran egg elicitors can also induce the production of oviposition-induced plant volatiles (OIPVs) enabling the plants 7) to attract egg or larval parasitoids, that upon locating their hosts, inject their own eggs and kill the lepidopteran instars to feed their off-spring (Tamiru et al., 2011; Fatouros et al., 2012; Cusumano et al., 2015; Ponzio et al., 2016) or 8) insectivorous birds (Mrazova et al., 2019). In addition, OIPVs can also prime 9) neighboring plants (Mutyambai et al., 2016; Guo et al., 2019).

Beyond the ability of adult Lepidoptera to perceive and process plant cues, thus modifying their oviposition behavior, there is a deep gap in the knowledge of possible egg counteradaptations used to overcome the bulk of oviposition-induced plant defenses. More information is available on the diversity of plant responses elicited by egg-laying (**Figure 1**), which are reviewed hereafter by narrowing the discussion to the most recent literature.

## The Plant Side: Local and Systemic Responses to Lepidopteran Egg Deposition

Insect oviposition on a host plant represents a particularly high risk for future herbivore attack and can enable plants to respond even before the actual damage occurs (Hilker and Fatouros, 2016).

### Egg-Induced Direct Plant Responses

Plant defense strategies can directly target insect eggs through desiccation, dropping, and crushing, eventually leading to egg mortality (Hilker and Fatouros, 2015). Egg deposition of some herbivores can induce reactions in plants that resemble a hypersensitive-like response (HR). This mechanism usually activated by pathogens causes rapid cell death and results in the formation of necrotic plant tissue, leading to the isolation of the pathogens from healthy tissues (Lam et al., 2001). The formation of leaf necrosis in response to insect egg deposition leads to the detachment of eggs from leaves or to their desiccation. This process was observed for the first time in *B. nigra* in which a necrotic zone develops 24 hours after *Pieris rapae* oviposition; in 72 hours, the eggs dry out and often fall off (Shapiro and DeVay, 1987). HR-like necrosis following *P. brassicae* egg-laying was observed also in different plants belonging to the Brassicaceae family (Pashalidou et al., 2015; Griese et al., 2019). Probably a decrease of humidity due to cell apoptosis underneath the oviposition site can cause a release of water out of the eggs eventually leading to their shrinking (Fatouros et al., 2014; Griese et al., 2017).

Recently, Griese and colleagues (2017) demonstrated that the effectiveness of HR-like necrosis in *B. nigra* varies with plant genotype, plant individual, and the type of egg-laying behavior (singly or clustered). Egg bunching could be a strategy to overcome plant defenses by keeping eggs from dehydration. Thus, in *P. brassicae*, egg clusters are more effective to avoid egg-killing compared to the single egg deposition, while the plant genetic background defines the likelihood and severity of HR under natural conditions. The authors hypothesized that the formation of HR-like necrosis evolved as a defensive trait against lepidopteran specialists of brassicaceous plants (Griese et al., 2017). This hypothesis was tested by the same research group who showed that elicitation of HR-like necrosis is specific to the Pierinae subfamily, whose species are adapted to brassicaceous host plants. Non-brassicaceous feeding species were not shown to induce HR-like necrosis (Griese et al., 2019).

Localized cell death was also observed in *Arabidopsis thaliana* after *P. brassicae* egg-laying (Little et al., 2007; Gouhier-Darimont et al., 2019); however, the response in this plant species is less strong and specific compared to *Brassica* spp., being *A. thaliana* not a foodplant for these butterflies (Harvey et al., 2007).

FA second morphological plant response to insect eggs is neoplasm formation (Petzold-Maxwell et al., 2011; Geuss et al., 2017). This process consists of the growth of a new plant tissue (callus) below insect eggs, which may lead to egg detachment (Petzold-Maxwell et al., 2011). Neoplasm formation in combination with HR-like necrosis was shown to be an egg-killing response in several solanaceous species. Oviposition by a specialist moth *Heliothis subflexa* induced such responses in two ground-cherry species (*Physalis* spp.) (Petzold-Maxwell et al., 2011).

More recently, Geuss et al. (2017) demonstrated that *Solanum dulcamara* responds to *Spodoptera exigua* eggs with the formation of neoplasms and chlorotic tissue. The accumulation of high levels of ovicidal hydrogen peroxide at the oviposition site leads to egg-killing.

### Egg-Induced Indirect Plant Responses

FOviposition can induce changes in the leaf chemistry (Fatouros et al., 2008) or trigger the production of volatile organic compounds (VOCs) called OIPVs (oviposition-induced plant volatiles) acting as synomones, i.e. indirectly harming eggs or imminent herbivores through the attraction of their natural enemies.

Alterations of the leaf chemistry composition that can be perceived by egg parasitoids after landing have been demonstrated in several crops and wild species following lepidopteran and hemipteran oviposition (Fatouros et al., 2005; Fatouros et al., 2008; Conti et al., 2010). For example, higher quantities of tetratriacontanoic acid and lower quantities of tetracosanoic acid (two important components of the epicuticular wax) were found in *A. thaliana* leaves after *P. brassicae* oviposition. These changes in molecule levels were shown to be fundamental in retaining *Trichogramma* wasps to egg-infested leaves (Blenn et al., 2012).

Lepidopteran egg-laying does not cause obvious damages in plants (Tamiru et al., 2011; Fatouros et al., 2012), as it occurs in other herbivores, e.g. leafhoppers and beetles (Hilker et al., 2002). Therefore, in contrast to the significant or qualitative changes prompted by herbivory in the plant volatile blends, OIVPs involve primarily quantitative variations (Hilker and Fatouros, 2015), yet effective in attracting parasitoids of lepidopteran eggs and larvae and even insectivorous birds (Mäntylä et al., 2018). This has been demonstrated on egg-laden black mustard (*B. nigra*) and landrace maize varieties (*Zea mays*), which induce emission of volatiles able to attract *Trichogramma* egg parasitoids (Tamiru et al., 2011; Fatouros et al., 2012; Cusumano et al., 2015; Ponzio et al., 2016).

While the ability of “warning” neighboring plants by means of volatile compounds released against herbivorous attacks is known to occur in various species (Heil and Ton, 2008), the existence of priming by OIPVs has been proven only recently. The study by Mutyambai and colleagues (2016) demonstrated that OIVPs released from the maize landrace ‘Nyamula' are able to attract the parasitoid wasp (*Cotesia sesamiae*) of the stem borer, *Chilo partellus*. These OIVPs also trigger an indirect defense response in neighboring conspecific plants even when they are not directly exposed to eggs. Among the volatiles released from maize following *C. partellus* egg-laying or exposed to OIPVs, the authors detected a strong emission of (E)-4,8-dimethyl-1,3,7,nonatriene (DMNT), a key homoterpene known as a mediator of herbivore-parasitoid system, with other terpenoids (limonene and myrcene), phenylpropanoids (methyl salicylate) and decanal, compounds often involved in tritrophic interactions.

Egg deposition or treatment with elicitors did not show particular effects in commercial standard maize hybrids, indicating a possible loss of defense traits in plants subjected to artificial selection and breeding (Mutyambai et al., 2016; Tamiru et al., 2017) and, as in the case of HR-like necrosis in *B. nigra* (Griese et al., 2017), highlighting the role of plant genotype in defense mechanisms.

The role of OIPVs in inducing defenses in neighboring plants was not only demonstrated in maize, but also in two clones of *Populus* egg-laden by the moth pest, *Micromelalopha sieversi* (Guo et al., 2019). The authors observed that neighboring plants are able to activate defense responses triggered by the release of volatiles cues (3-carene and β-pinene) from oviposited plants, including the production of VOCs aimed to prevent egg-laying.

Eggs laid by herbivorous insects on a plant leaf indicate that larval feeding will soon occur. Recent studies have demonstrated that, in addition to the enhanced attraction of larval parasitoids (e.g., Pashalidou et al., 2015), “early herbivore alert” responses can also increase plant defense against future herbivory (revised by Hilker and Fatouros, 2015; Hilker and Fatouros, 2016). While a few studies indicate that insect egg deposition may suppress plant anti-herbivore defenses (Bruessow et al., 2010; Peñaflor et al., 2011), additional studies comparing plant responses to egg-laying by several generalist and specialist insects are necessary to elucidate the mechanisms involved in this process.

### Defense Pathways and Gene Expression

It is well known that elicitors (see below), associated to egg deposition, trigger electrical signals and change Ca^2+^ homeostasis. This is subsequently followed by downstream defense responses regulated through hormone signaling pathways, whose jasmonic acid (JA) and salicylic acid (SA) are the major players involved (Reymond, 2013). Both the individual hormones and their crosstalk play an essential role in fine-tuning defense responses to specific herbivores (Proietti et al., 2018).

The induction of the JA pathway by herbivore-associated elicitors has been extensively reported; however, there is no clear evidence that the JA-pathway is induced by insect egg deposition.

The response to oviposition by *P. brassicae* on *Arabidopsis* or *Brassica* spp., where eggs are laid on the leaf surface without any damage, appears mainly controlled by SA signaling pathway. In *Arabidopsis* plants, SA accumulated at high levels underneath *Pieris* eggs and several SA-responsive genes were upregulated by egg-laying also in systemic leaves (Hilfiker et al., 2014; Bonnet et al., 2017). These responses were absent in some *Arabidopsis* mutants lacking the SA-signaling pathway (Gouhier-Darimont et al., 2013). This defense mechanism is similar to the response triggered by pathogens (Gouhier-Darimont et al., 2013).

It is clear that lepidopteran oviposition induces different morphological, physiological, and chemical responses in plants that are strongly correlated to the variation in gene expression levels. The first study of *P. brassicae* egg-induced transcriptional changes performed with *Arabidopsis* whole-genome DNA microarrays showed the up-regulation of several defense-related genes, including some regulating cell death and innate immunity, and others involved in stress responses and in secondary metabolite biosynthesis (Little et al., 2007). More recently, a transcriptome comparison of *Arabidopsis* feeding-damaged leaves, with and without prior oviposition, revealed the up-regulation of *PR5*, a gene involved in SA-signaling, an increase in SA levels and flavanol accumulation in egg-laden but not yet damaged plants (Lortzing et al., 2019). Also Geuss et al. (2017) showed that feeding larvae of *S. exigua* induced an increase in *S. dulcamara* resistance, by changing its transcriptional and metabolic responses at both the local and systemic level. In particular, genes involved in phenylpropanoid metabolism were upregulated in previously oviposited plants, suggesting a crucial role of these molecules in oviposition-primed plant resistance.

Moreover, a study conducted on maize landrace Braz1006 demonstrated that both *C. partellus* egg deposition and a treatment with an elicitor that mimics herbivory can induce the up-regulation of the gene coding for the terpene synthase TPS23, which catalyzes the final step in the biosynthetic pathway of (E)-caryophyllene, an important signaling molecule involved in plant-herbivore interactions (Tamiru et al., 2017).

### Egg-Derived Elicitors

During oviposition, insects produce a vast range of substances from the ovary and accessory glands, which can act as **elicitors** of the above-mentioned plant defenses.

These secretions can provide eggs with protection against biotic and abiotic threats, facilitate their deposition (lubrification) or their substrate attachments. Beyond being found on the egg surface or at the plant-egg interface, bioactive compounds can also be found within the egg. Yet, the role of the inner compounds in eliciting plant responses seems unlikely due to the presence of physical barriers (e.g. eggshell, adhesive glue) hindering the access to plant cell targets (Hilker and Fatouros, 2015). Bruessow et al. (2010) suggested that elicitors should be found within the eggs, in the embryo, as no reaction was observed when empty *P. brassicae* eggshells were applied at the leaf surface. However, the lack of any response could be due to external egg elicitor inactivation (instead of their absence) that occurs in the period between deposition and hatching event (Fatouros et al., 2015).

Experiments conducted with crushed egg extracts (EE) mimicked the response observed upon egg-laying in *A. thaliana* (Little et al., 2007). Using an *Arabidopsis* transgenic line containing the promoter of the egg-induced gene *PR1* coupled to the β-glucuronidase (GUS) reporter gene, Little et al. (2007) demonstrated that the application of soluble *P. brassicae* EE activates GUS and triggers plant responses. Similar results were obtained when EE from distantly related insects, either generalists or specialists, were applied to *A. thaliana* transgenic plants.

Although a very few compounds have been isolated, benzyl cyanide was identified as a molecule responsible for surface chemical changes induced by *P. brassicae* oviposition on *Brassica oleracea* var. *gemmifera*. The application of this male-derived anti-aphrodisiac mimicked the egg-induced arrestment of *Trichogramma brassicae* (egg parasitoids) in *B. oleracea* and *Arabidopsis* leaves (Fatouros et al., 2005; Blenn et al., 2012). Moreover, *P. rapae* females receive methyl salicylate and indole as anti-aphrodisiac compounds during mating. When applied onto the leaf, indole induced changes in the foliar chemistry that arrested *T. brassicae* wasps (Fatouros et al., 2009).

Besides the extensive research on plant-insect interactions and although it is generally assumed that plants detect elicitors through cell-surface receptors, to date, no such protein has been isolated and described. Following different attempts, in 2019, Gouhier-Darimont and co-workers identified an important component of *A. thaliana* perception system for insect eggs, LecRK-I.8, a L-type lectin receptor kinase. This protein seems to play a key role in early signal transduction steps by controlling several responses to *P. brassicae* egg-laying. The authors demonstrated that a lipidic fraction from *P. brassicae* eggs triggers localized cell death and that this response is significantly attenuated in *lecrk-I.8* mutant plants, suggesting that LecRK-I.8 is involved in the sensing of an egg-derived lipidic compound (Gouhier-Darimont et al., 2019).

## A Third Player: Egg-Associated Microorganisms

Symbiotic bacteria play a pivotal role in the development and survival of their insect hosts, providing a full array of molecules for digestion, detoxification, and defense against pathogens (Douglas, 2015). There is still a scant knowledge on Lepidoptera-associated microbiomes, because the majority of studies is (i) merely descriptive, (ii) focused on single bacterial taxon, (iii) a few butterfly/moth species have been extensively surveyed, or (iv) only rarely endosymbionts have been compared across different developmental instars (Di Salvo et al., 2019; Gao et al., 2019; Szenteczki et al., 2019). Nevertheless, an increasing number of experiments provide evidence for a crucial function of microbes in basic physiological processes of Lepidoptera (Paniagua Voirol et al., 2018), e.g. through the modulation of salivary elicitor biosynthesis (Wang et al., 2018).

Since data gathered until now suggest a remarkable diversity of (gut) microbiomes across diets and stages, it is questioned whether Lepidoptera harbor resident beneficial microbes or more likely acquire from food and/or environment a plastic microbial community, which favors them under changing conditions (Hammer et al., 2017). If confirmed, this scenario implies that eggs might not serve as the means for achieving the vertical transmission of core gut microbiomes, but only of other microbial symbionts. The inherited microbes could also be present on the egg surface and transferred by eggshell ingestion to newly hatched larvae (Duplouy and Hornett, 2018), but their characterization and function are completely lacking.

The occurrence of egg-associated bacteria has been reported for a few species including *Manduca sexta*, *Rothschildia lebeau*, *Spodoptera littoralis*, and *Lymantria dispar* (Paniagua Voirol et al., 2018), but there are no insights about potential roles of egg-associated bacteria in eliciting plant responses.

## Conclusion

Egg-laying patterns are the outcomes of complex evolutionary dynamics shaped by physical, physiological, and ecological characteristics of the host plants. Although plant responses to both eggs and herbivores have been extensively explored (Hilker and Fatouros, 2015; Schuman and Baldwin, 2016), only a few studies have dealt with herbivore counteradaptations (Karban and Agrawal, 2002) and even less with egg defensive/offensive traits (Bruessow et al., 2010; Peñaflor et al., 2011). However, an increasing number of insights suggests that (i) the female ability to identify plants with inadequate plant defenses could be an evolutionarily advantageous strategy and (ii) the biochemical apparatus of plants could be subverted by egg compounds to inhibit or lower the LHP defenses against the incoming larval instars.

Unfortunately, the advance of this research is constrained by the lack of upstream knowledge about basic mechanisms fostering the specificity of plant responses. The latter are likely based on still undiscovered egg-associated compounds (elicitors) and their plant receptors, which therefore should be among the first issues to be tackled.

## Author Contributions

CB and FB led the writing of the manuscript to which all authors contributed critically and gave final approval for publication.

## Conflict of Interest

The authors declare that the research was conducted in the absence of any commercial or financial relationships that could be construed as a potential conflict of interest.
